# Preparation of solution processed photodetectors comprised of two-dimensional tin(ii) sulfide nanosheet thin films assembled *via* the Langmuir–Blodgett method†

**DOI:** 10.1039/d1ra04470b

**Published:** 2021-08-06

**Authors:** Kane Norton, Janet Jacobs, Joseph Neilson, David Hopkinson, Mohammad Z. Mokhtar, Richard J. Curry, David J. Lewis

**Affiliations:** Department of Materials, University of Manchester Oxford Road Manchester M13 9PL UK david.lewis-4@manchester.ac.uk; Photon Science Institute, Department of Electrical and Electronic Engineering, University of Manchester Oxford Road Manchester M13 9PL UK richard.curry@manchester.ac.uk

## Abstract

We report the manufacture of fully solution processed photodetectors based on two-dimensional tin(ii) sulfide assembled *via* the Langmuir–Blodgett method. The method we propose can coat a variety of substrates including paper, Si/SiO_2_ and flexible polymer allowing for a potentially wide range of applications in future optoelectronic devices.

## Abstract 2

Norton *et al*. report the manufacture of fully solution processed photodetectors based on two-dimensional tin(ii) sulfide assembled *via* the Langmuir–Blodgett method.

## Graphical abstract

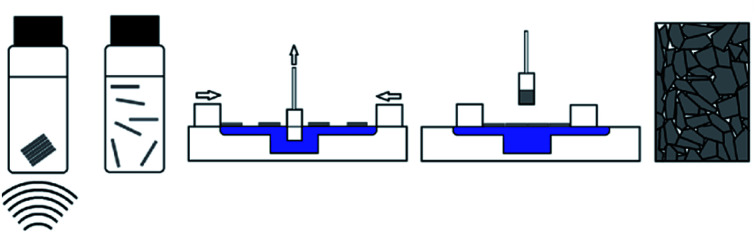

Two-dimensional (2D) materials are condensed matter solids formed of crystalline atomic layers held together *via* weak van der Waals forces.^1^ They have a wide range of applications including use as channel materials in transistors,^2^ absorber layers in solar cells,^3^ light emission,^4^ energy storage^5^ and drug delivery^6^ among others. 2D materials often have different properties from their bulk counterparts such as increased strength^7^ and electrical conductivity.^8^ 2D semiconductors may exhibit a change in electronic states from confinement in 1D.^9^ Thin films are often required for the creation of devices from nanomaterials for practical applications and can often be made into flexible devices such as thin film solar cells^10^ or photodetectors.^11,12^ Thin film solar cells in particular have several advantages over conventional solar cells including lower materials consumption and are lightweight, yet have the potential for high power conversion efficiency.^10^

Many of the two-dimensional materials produced thus far have been derived from mechanical exfoliation, where Scotch tape or an equivalent is manually used to remove single crystalline layers from a bulk van der Waals solid followed by transfer to a substrate. Whilst this method in general produces extremely high quality crystalline atomic layers,^13^ and is therefore often used to produce prototype devices, it inherently lacks scalabilty. In order to address the problem of mass manufacture of two dimensional materials, liquid phase exfoliation (LPE) was introduced as a cost effective method for producing two dimensional nanomaterials^14^ with the possibility of 100 L scales being produced and production rates up to 5.3 g h^−1^ demonstrated by Coleman *et al.* with both NMP and aqueous surfactant solutions utilised.^15^ This method also does not require the high temperatures needed for methods such as CVD^16^ or transfer between the growth and final substrates. Liquid phase exfoliated nanomaterials are also directly processable from solution.^15^ Furthermore, LPE has been shown to be effective for the production of a wide range of 2D materials such as graphene,^15^ transition metal dichalcogenides^17^ and monochalcogenides such as SnSe.^18^

Tin(ii) sulfide (SnS) is a van der Waals solid with a puckered *ab* structure consisting of alternating Sn and S atoms, and is isostructural and isoelectronic with black phosphorus.^19^ The bulk material has attracted interest due to its indirect band gap energy of 1.07 eV,^20^ similar to bulk silicon at 1.14 eV. This band gap energy for SnS is useful for applications such as photodetection^21^ and due to its higher theoretical Shockley–Queisser efficiency limit (24%) for solar cells.^22^ The liquid phase exfoliation method established by Coleman *et al.* enables nanosheets to be separated from the bulk into solution utilising matching surface energies of the material and solvent.^23^ Liquid phase exfoliation of SnS was first reported by Lewis *et al.* it was established that as layer number reduced, band gap energy increased, and by tuning layer number the onset of photon absorption can be tuned over the near infrared^23^ to visible range.^24^ Overall, LPE is capable of creating large quantities of nanosheets, with potential for industrial scale production. Liquid phase exfoliated SnS has, for example, recently been used in the creation of photoelectrochemical systems with strong stability under both acidic and alkali conditions.^25^ Many of the functional devices produced thus far have been derived from micromechanical exfoliation and manual nanomanipulation. A far more elegant solution to producing functional devices is to assemble them from solution, for example Kelly *et al.* recently reported a transistor based on exfoliated WSe_2_ nanosheets.^2^

The Langmuir–Blodgett method involves the use of a trough with a layer of water and controllable barriers to compress the film. Nanomaterials in solution are added to the surface of the water and spread evenly to reduce their surface energy,^26^ often by using a low surface tension spreading solvent such as chloroform.^27^ The surface pressure is measured as the film is compressed with the substrate being withdrawn when the film becomes solid.^28^ The Langmuir–Blodgett method has the advantages of large area deposition and improved control of the film at the nanoscale in comparison to vacuum filtration as well as the advantage of requiring no volatile solvents in comparison to liquid–liquid assembly methods. The use of movable barriers also allows for greater film compression.^26^

This method has been used to assemble large scale films of exfoliated MoS_2_ by Zhang *et al.* MoS_2_ was exfoliated using *n*-butyl lithium followed by solvent exchange. MoS_2_ was deposited onto the water surface using a 1 : 1 mix of DMF and dichloroethane. Substrates up to 130 cm^2^ were coated with a surface coverage of 85–95%.^26^ Collapse mechanisms of MoS_2_ Langmuir films have also been studied^29^ alongside MoS_2_ deposition on the surface of water with an upper hexane layer.^30^ Graphene films have also been prepared using the Langmuir–Blodgett method.^31^ The Langmuir–Blodgett method has been used for the assembly of organo-clay hybrid films *via* the coating of octadecylammonium chloride in a 4 : 1 chloroform : ethanol solution onto a 2D nanoclay liquid phase exfoliated film using an electrospray method.^32^ A solvent mix of chloroform and NMP has also been utilised for the deposition of nanosheet films.^33^ Recently the Langmuir–Blodget method has been used for the assembly of unmodified clay nanosheets,^34^ Ti_3_C_2_Tx MXene nanosheet films for the removal of Cr(vi) and methyl orange from an aqueous environment^35^ as well as for the growth of rGO wrapped nanostructures for use in electrocatalysts.^36^

Given the chemical similarity of the basal planes of inorganic 2D materials, we hypothesised that the assembly of group IV–VI nanomaterials such as SnS should also be possible at the air water interface. Due to their interesting semiconducting and properties described, it should also be possible to produce prototype optoelectronic devices from a fully solution processed pathway. In this paper we now communicate a methodology to assemble thin films comprised of 2D SnS nanosheets using the Langmuir–Blodgett technique (Scheme 1a). We report the use of these films in simple photodetectors. This represents a scalable methodology to produce fully solution processed devices based on 2D materials.

**Scheme 1:**
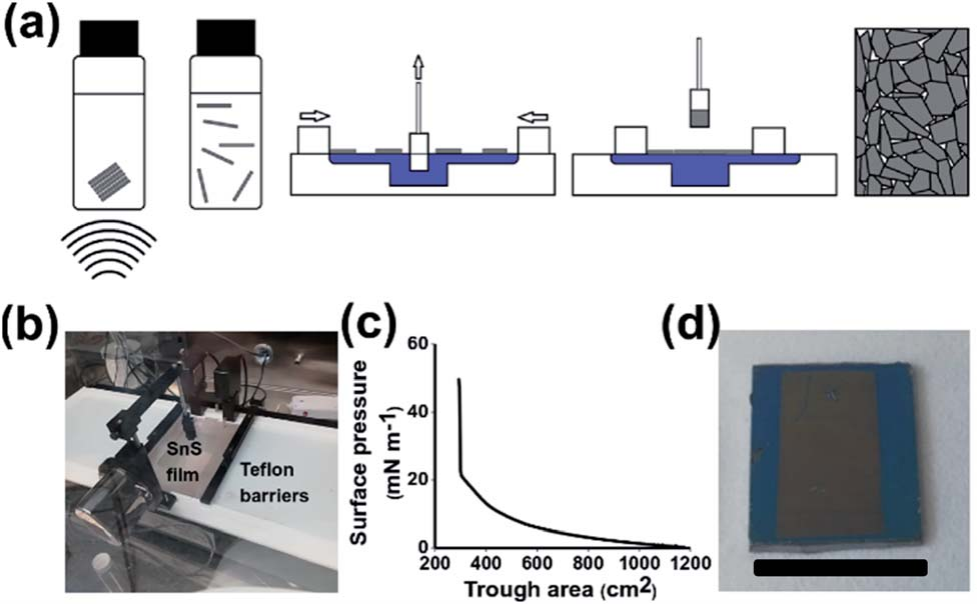
Preparation of SnS nanosheet thin films *via* the Langmuir–Blodgett method. (a) Cartoon of Langmuir–Blodgett film preparation. (b) Image of Langmuir–Blodgett trough with compressed SnS film. (c) Surface pressure profile during film compression. (d) Image of sample prepared on Si/SiO_2_ substrate with edges masked (scale bar 1.5 cm).

Scheme 1(a) shows the step by step process of film preparation. Firstly, bulk SnS is broken down by liquid phase exfoliation from the bulk material to produce a stable dispersion of crystalline nanosheets. Characterisation of the exfoliated nanomaterials was undertaken using atomic force and electron microscopy yielding average sheet dimensions of 23.9 nm height × 224 nm longest side length (Fig. S1†). The nanosheets were then deposited onto the water air interface. The film is then compressed whilst an immersed substrate is withdrawn, leading to the creation of a densely packed nanosheet film. Scheme 1(b) shows that SnS can be successfully deposited on the water–air interface *via* the addition of chloroform as a spreading solvent, as shown previously with other Langmuir based films.^27^Scheme 1(a) shows a z-type deposition of SnS as the hydrophilic glass and Si with a 300 nm oxide layer is withdrawn through the film at 1 atm pressure. The film compression occurred at a rate of 5.88 cm^2^ s^−1^. No further treatments were performed to change the hydrophilicity of the substrates, the oxide layer present was sufficient to provide hydrophilicity to the substrate.^37^Scheme 1(c) shows a gradual increase in surface pressure as the area was decreased from 1175 cm^2^ to 298 cm^2^ before a sharp increase in pressure, indicating the film has reached full compression. The sharp increase in surface pressure during compression is common in Langmuir–Blodgett assembled films of nanomaterials.^38^ In response to compression the surface pressure profile in Scheme 1(c) rises rapidly until it reaches a maximum due to the size of the sheets and the potential difficulty in sliding over each other compared to polymers or smaller nanomaterials. Scheme 1(d) shows that the film is capable of being coated onto Si/SiO_2_ with a mask defining the areas covered.

We characterised the resulting structural and electronic properties of the thin film of SnS nanosheets deposited *via* the Langmuir–Blodgett method using a range of techniques. Fig. 1(a) shows a height profile AFM image of a film edge with an average on-film roughness (*R*_a_) of 31.9 nm and an average film thickness of 78.6 nm (Fig S3† provides an additional film profile). Previous work on Langmuir–Blodgett deposition has produced thinner films. The use of high centrifugation speeds yielded 7 nm thick films for a single deposition^31^ whilst the use of lithium ion intercalation before exfoliation enabled film thicknesses of under 2 nm per layer to be realised.^26^ The average film thickness is above the average sheet thickness, suggesting that the film is made up of overlapping flake multilayers. However, the thickness of the films is significantly lower than those grown *via* chemical bath deposition (*e.g.* 290 nm (ref. 39)) indicating that thinner films can be produced compared to chemical bath methods, and potentially at a much lower cost than methods such as CVD. Images of the film morphology in plan view SEM (Fig. 1(b)) suggest no notable alignment of the nanosheets in the lateral dimension as the film is formed and deposited (see Fig S4† for statistical analysis of sheet angle measurement). The coverage of the film is 94.6% as determined by image thresholding using imagej software to determine the area left uncovered. This gives a coverage of 0.0142 gm^−2^ as calculated from average thickness, SnS density and % coverage of the substrate. Preliminary SEM results also suggest that the Langmuir–Blodgett method is effective at coating SnS onto a variety of substrates including polyolefin films (Parafilm®), aluminium foil and paper (Fig S6†). We also probed the structure of the thin films by powder X-ray diffraction (XRD). After exfoliation and film assembly, the diffraction peak associated with the (400) of SnS is still the most intense reflection but is characterised by a much larger FWHM compared to that of bulk SnS under the same recording conditions (0.442° ± 18.5% compared to 0.175° ± 5%). This indicates a successful breakdown of the crystal structure and thinning of the material in the (400) plane during exfoliation due to the reduction in long range order^40^ (reflections for bulk SnS are assigned to orthorhombic SnS and indexed in Fig S2†). The lack of any additional peaks indicates that there has not been any significant degradation of the material to the corresponding oxide which is in agreement with previous works.^24,25^ The reflections at 88° and 94° are unlikely to be from crystalline silicon^41^ due to the thick oxide layer and low angle of incidence used. We tentatively ascribe these peaks to the 3,0,−3 and 3,2,4 peaks for SnS.^41^ However a confident assignment of this reflection requires further studies.

**Fig. 1:**
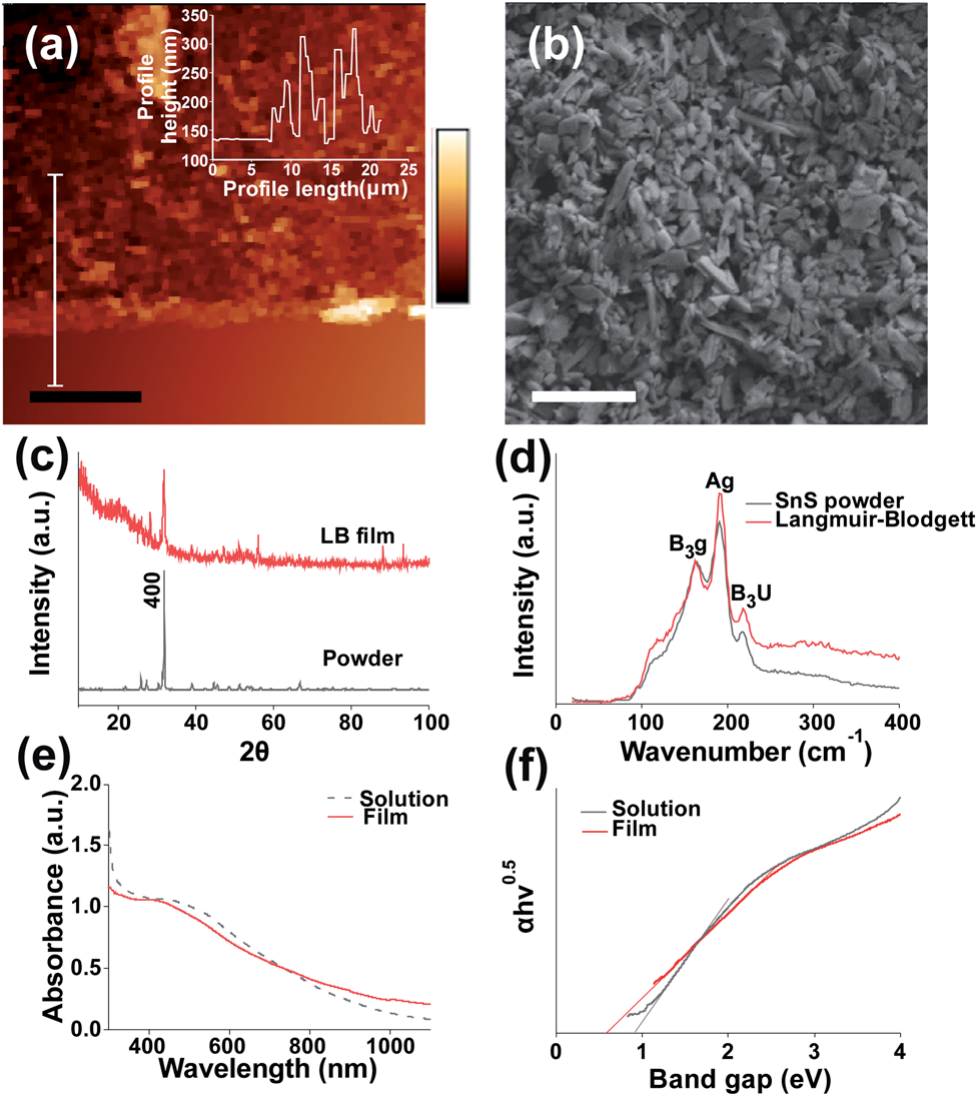
Structural characterisation of SnS nanosheet thin films assembled by the Langmuir-Bllodgett method. (a) AFM image of LB assembled SnS film edge. Inset film profile, scale bar = 10 μm. (b) SEM image of LB assembled film on Si/SiO_2_ at 3 kV using secondary electron imaging, scale bar = 1 μm. (c) XRD pattern of coated film and bulk SnS powder, (additional peaks labelled in Fig. S2†). (d) Raman spectra and for bulk and Langmuir–Blodgett assembled SnS nanosheets. (e) UV-Vis spectra of SnS suspension and deposited SnS film on glass (f) Tauc plot of SnS solution and film.

We also characterised the optical properties of the nanosheet thin films using Raman and UV-Vis-NIR absorption spectroscopy. No shifts in the Raman peak positions B_3_g, Ag and B_3_u from bulk SnS to Langmuir–Blodgett film were observed. The broad feature at around 300 cm^−1^ for the LB film may potentially be due to SnS_2_ and Sn_2_S_3_ impurities.^42^ It is predicted that due to the lower density compared to SnS^43^ the impurities may increase in concentration compared to the bulk after centrifugation. These impurities may have significant effects on the efficiency of the devices produced.^44^

A shift in peak positions is typically observed in nanomaterials which exhibit quantum confinement,^45^ this occurs at 14 nm for SnS.^46^Fig. 1(e) shows a UV-Vis spectra from which the absorption coefficients at fixed wavelengths may be obtained, for 350 nm, 405 nm, 450 nm, 500 nm, 600 nm and 800 nm the values obtained were: 2.26 × 10^5^ cm^−1^, 2.21 × 10^5^ cm^−1^, 2.16 × 10^5^ cm^−1^, 2.04 × 10^5^ cm^−1^, 1.67 × 10^5^ cm^−1^ and 1.05 × 10^5^ cm^−1^ respectively, this matches well to the absorption coefficients of SnS in literature (greater than 10^4^ cm^−1^).^47^ It also suggests there may be a greater response at shorter wavelengths.

Fig. 1(f) shows a band gap of 0.92 eV for the exfoliated SnS in NMP which is below the expected value of 1.07 eV (ref. 20) although lies within the reasonable error introduced by the use of Tauc plots.^48^ The band gap also matches well with SnS exfoliated in NMP in previous work.^24^ The band gap of the film appears to change from nanosheet suspension to film in 1(f). This has been observed previously for Langmuir–Blodgett^49^ and other deposited films. It has also been observed that apparent decreases in band gap may occur due to the presence of scattering artefacts within films of nanoscale objects.^50^

We then produced simple prototype photodetectors *via* the printing of Ag nanoparticles to form interdigitated electrodes on top of the SnS nanosheet film. Additionally, SnS films were deposited onto lithographically defined Au interdigitated electrodes for characterisation and referencing to the printed devices.

Previously SnS photodetectors have been created *via* methods such as electron beam deposition,^51^ thermal evaporation^52^ and chemical bath deposition.^53^ The Langmuir–Blodgett method allows SnS to be directly processed into a film from a liquid phase exfoliated solution, allowing them to be produced cheaply and with the potential for scalability.

Inset to Fig. 2(a) is an image of an interdigitated Ag electrode SnS photodetector device with an area of 6.4 × 10^−5^ m^2^. The electrodes can be clearly identified with an average spacing of 99 μm, and an average RMS edge roughness value of 1.89 μm (determined for individual contact lines using the imageJ ‘analyze_stripes’ plugin^54^ (Fig S7†)). Fig. 2(a) shows an increase in the slope of the *I*–*V* curve in the third quadrant indicating a reduction in resistance under 1 sun illumination (1000 W m^−2^) with the AM1.5 spectrum. No short circuit current under illumination was observed indicating that the device functions as a photoconductor. The non-linear response upon negative biasing is due to initial trap filling which once equilibrium has been reached results in linear device operation. Previously it has been shown that silver diffusion into SnS has an interstitial doping effect, neutralising defect states and lowering the film resistivity.^55,56^ It is also possible that the Ag ink morphology and the concentration of nanoparticles in the ink may play an effect on the device properties.^57^ A resistivity of 2.85 × 10^6^ Ω sq^−1^ was obtained for the device which is significantly higher than SnS films prepared by physical vapour deposition (250 Ω sq^−1^),^58^ likely due to poor carrier mobility between flakes.

**Fig. 2:**
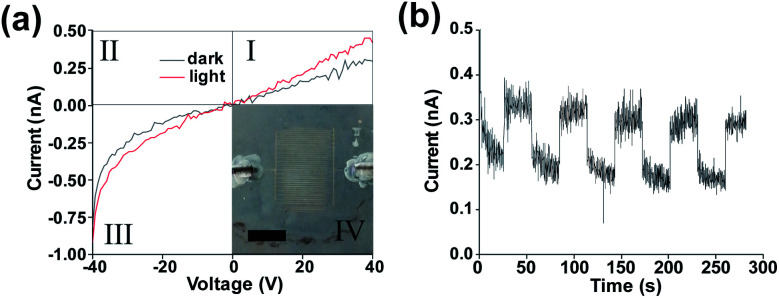
(a) IV curves of printed contacts SnS device under darkness and AM1.5 illumination with inset photograph of pseudo Langmuir–Blodgett device with printed Ag contacts scale bar 5 mm. (b) Device under +40 V bias under fixed darkness/illumination cycle.

Fig. 2(b) indicates that a clear response is present under illumination when an external bias is applied (giving a field strength of 0.4 V μm^−1^). Closer inspection shows a fast and slow decay component following the illumination being blocked. This biexponential decay indicates the capture of trapped carriers and the presence of trap states within the device.^59,60^ This again supports the photoconductive nature of the device operation with a rise time of ∼0.22 s and a fall time of ∼2.83 s,^61^ both being longer than the shutter closing/opening time of 3.7 ms (which was considered negligible). The rise time is the time taken to get from 10% to 90% of the light current with the fall time being the time taken from 90% of the light current to 10%.

Previous work performed by Jiang *et al.* has shown a slow fall time in Ag/SnS photoconductor devices arising from carrier trapping.^62^ Similarly, in our devices the large rise time may also be due to the presence of a high trap density which must be filled upon light exposure.

The mean dark current is 2.78 × 10^−10^ A with a standard deviation of 2.02 × 10^−11^ A. The mean light current was found to be 3.92 × 10^−10^ A with a standard deviation of 4.03 × 10^−11^ A. A poor signal to noise ratio appears to be present within the device, possibly due to the large number of SnS nanosheets involved in charge carrier transit, leading to a low signal, hence a low signal to noise ratio. The noise could be reduced *via* surface passivation^63^ or the use of a diode like structure to reduce leakage current under reverse bias.^64^ A low responsivity of 2.00 × 10^−9^ A W^−1^ ± 1.5 × 10^−10^ A W^−1^ was found for energies above the band gap energy of 0.6 eV for the deposited film.

The low responsivity may be due to poor bridging between individual SnS nanosheets and the poor transport of holes between adjacent flakes (hopping) relative to the higher mobility within each flake.^65^ There are potentially hundreds of nanosheets between the contacts as determined by the average length obtained (Fig S1†). To confirm that the optical response was due to the presence of the SnS a reference device was tested (without SnS deposition, Fig S8†) with no photoresponse observed. Despite the low responsivity, it is notable that the SnS devices fabricated are one of the few examples of a thin film photodetector device based on 2D materials requiring only solution processing at ambient temperature and atmospheric pressure.

To demonstrate that the observed behaviour originates from the photoresponse of the SnS flakes a second device was fabricated by pseudo Langmuir–Blodgett deposition on to lithographically defined Au interdigitated electrodes (15 μm separation) on fused silica (inset Fig. 3(b)). This enabled us to remove any effect of photoinduced Ag migration from the observed behaviour as well as eliminating the issue of potential printing irregularities. Fig. 3(a) shows that the devices display a similar photoresponse to the devices with printed Ag electrodes when exposed to modulated AM1.5 illumination. The dark current remains similar at ∼0.3 nA, though during illumination the current is higher (0.7 nA *vs.* 0.4 nA). This increase directly correlates to the higher electric field strength (0.66 V μm^−1^*vs.* 0.4 V μm^−1^) between the interdigitated electrodes. The responsivity of the device was determined to be 1.79 × 10^−8^ A W^−1^, with a photoresponse rise and fall time of 0.77 s and 0.85 s respectively. The responsivity is lower than for photodetectors prepared by Guo *et al.*^66^ Improvements to the device to improve the responsivity could include methods to improve the lateral size of nanosheets such as intercalation.^67^ Other routes to improve the device may include doping^68,69^ or a change in architecture to a phototransistor type device.^70^ The removal of potential SnS_2_ and Sn_2_S_3_ impurities *via* methods such as annealing at 500 °C, 500 mbar pressure under argon or the use of higher quality starting material may also be a key route to improve the efficiency of the device.^42^

**Fig. 3:**
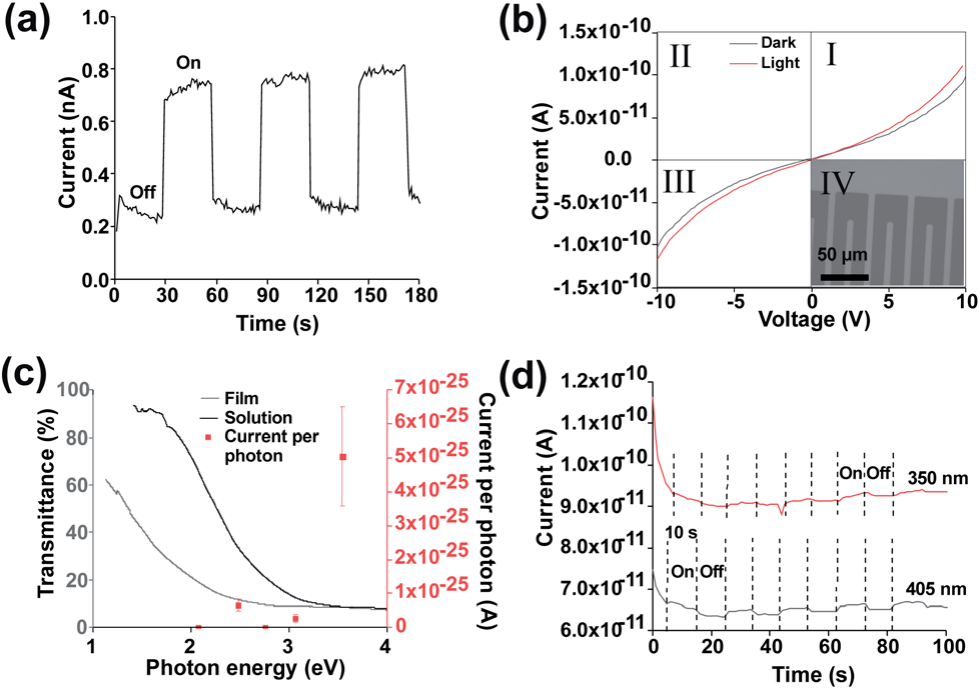
(a) Device under 30 s off, 30 s on solar simulator illumination at 1 sun and 10 V bias (b) IV curves under darkness and 350 nm illumination with inset optical microscopy image of contacts (c) monochromatic illumination responses under 10 V bias mapped onto UV-Vis transmission spectra (d) device response under fixed 10 V bias under 350 nm and 405 nm monochromatic illumination.

It is also noticeable that the level of noise present in Fig. 3(a) is reduced compared to that in Fig. 2(b), indicating that the Ag electrodes themselves (in addition to the SnS sheets) also affect the performance.

When exciting using AM1.5 illumination it is possible that thermal effects may be present which could give rise to the observed behaviour.

In order to demonstrate a true photoresponse monochromatic illumination was used to determine if illumination energies above the band gap generated a photocurrent response in the device. Fig. 3(b) shows a small response under 350 nm (3.54 eV) illumination. (IV curves for other wavelengths are available in Fig. S9†). Fig. 3(c) shows an increased response for 350 nm wavelength as determined *via* the IV curves. This increased response is likely due to increased absorption as shown in the UV-Vis spectra (Fig. 1e), the signal at longer wavelengths is difficult to observe due to the low responsivity. A higher response at lower wavelength has been observed previously for SnS.^53^

Fig. 3(d) shows that an increase in current is present under 350 nm and 405 nm illumination which can be cycled on and off. A rise and fall time of 1.09 of 1.44 seconds respectively was observed for 405 nm illumination. A light/dark current ratio of 1.03 was obtained under 405 nm. To account for noise the on and off section had their current averaged using origin software. A drift in current during measurement was observed, this was considered as the reason for the significant difference between the dark current for 350 nm and 405 nm. To further reduce noise surface passivation may also be used to improve the device properties.^63^ Alternatively, an increase in bias voltage or an increase in monochromatic illumination intensity may improve the signal: noise ratio though may risk damage to the device. A magnified off/on cycle for 405 nm is shown in Fig. S10.†

In conclusion, we report here a methodology for the assembly of 2D SnS nanosheets into thin films using the Langmuir–Blodgett method, and the testing of the films as prototype all-solution processed photodetectors. Tin(ii) sulfide was successfully exfoliated with an average sheet thickness of 33 nm with the average longest side length of 224 nm. A nanosheet based film was coated onto a variety of substrates *via* the Langmuir–Blodgett method with the addition of chloroform as a spreading solvent. The films were found to be polycrystalline with an average thickness of 78.6 nm with a high surface coverage up to 94.6% for an Si/SiO_2_ substrate. The films were found to be semiconductive with the ability to respond to light under bias as shown by AM1.5 and monochromatic illumination. Proof-of-concept photodetectors have been successfully produced. It was also confirmed that the response was due to the photoresponse as opposed to a heating effect. This deposition method could potentially be used to create a variety of SnS films using different exfoliated nanosheet sizes separated *via* cascade centrifugation as well as the potential for future flexible photodetector devices. Despite the low responsivity, large rise and fall times further work could allow the gain to be optimised. We also note that the use of the Langmuir–Blodgett trough is an easily scalable technology and could provide coatings over very large area substrates not only for photodetectors but for other devices such as thin film solar cells.

## Conflicts of interest

The authors declare no conflicts of interest.

## Supplementary Material

RA-011-D1RA04470B-s001
