# Supplementary material for: Preparation of solution processed photodetectors comprised of two-dimensional tin(ii) sulfide nanosheet thin films assembled via the Langmuir–Blodgett method
Source: RSC Adv. 2021 Aug 6;11(43):26813–9. doi: 10.1039/d1ra04470b (PMC9037678; doi:10.1039/d1ra04470b)
Supplement: RA-011-D1RA04470B-s001 [file RA-011-D1RA04470B-s001.pdf]

Electronic Supplementary Material (ESI) for RSC Advances.
This journal is © The Royal Society of Chemistry 2021

Supporting Information

Preparation of solution processed photodetectors comprised of two-dimensional tin(II) sulfide nanosheet thin films assembled via the Langmuir-Blodgett method

Kane Norton,[a] Janet Jacobs,[b] Joseph Neilson,[a] David Hopkinson,[a] Mohammad Z. Mokhtar,[a] Richard J. Curry[c] and David J. Lewis.*[a]

Experimental

Preparation of SnS nanosheet solution

0.5 g of tin (II) sulphide (Sigma Aldrich) was added to 25 ml of N-methyl pyrrolidone (NMP) (Sigma Aldrich). This was added to an elmasonic P water bath at 37 kHz at 30 % power (820 W across 4 horns) and sonicated for 24 hours 7.5 cm above the far left horn with 4 cm of the vial submerged. The solution was then centrifuged at 180 *g* for 45 minutes using a Multifuge X1 centrifuge, the solution was collected and re-centrifuged at 3040 *g* with the remaining solution being discarded, the sediment was redispersed in 25 ml fresh NMP via 5 minutes of sonication at 37 kHz, 30% power.

Preparation of the Langmuir-Blodgett film

Glass substrates and Si/SiO_2 substrates were cleaned by sonicating for 5 minutes in acetone, 5 minutes in isopropanol and 5 minutes in deionised water before air drying.
2 ml increments of NMP dispersed nanosheet solution were taken and diluted to 10% v/v using chloroform (VWR) then inverted to mix, the solution was added drop-wise onto de-ionised water in a Nima TR 8.1 Langmuir Blodgett trough. The coating and barrier speed used were 10 mm/min. The pressure was measured using a Wilhelmy plate. The film was coated onto cleaned Si/SiO_2 substrates, cleaned glass slides, filter paper (supplementary figure 4 c,d), aluminium foil (s.f 4b), parafilm (s.f. 4 a), a lacey carbon TEM grid and a section of polystyrene petri dish. The deposition was performed at 1 atm pressure. A schematic of the process is shown in figure 2 (a). A Si/SiO_2 wafer was masked using scotch tape before coating (figure 2 d). The samples were dried at 50 ^{o}C overnight in a vacuum oven.

Langmuir-Blodgett film:

A SnS film between 180 – 3040 *g* was coated onto a glass substrate via a pseudo Langmuir-Blodgett method. A glass dish 9cm diameter by 5.4 cm height was used in place of a full LB trough. The dish was filled to 4 cm with DI water. 1 ml increments of NMP exfoliated 180 – 3040 *g* nanosheets were diluted to 1/10 v/v in chloroform. This solution was added drop wise to the water surface using a glass dropper pipette until the film was no longer able to compress further. The glass substrate was lifted up through the film at a 45 degree angle using an Ossila dip coater. The film was dried on a hot plate at 50 ^{o}C.

Preparation of photodetector and reference devices:

Interdigitated contacts were printed onto a pseudo Langmuir-Blodgett film using a SIJ-3030 printer using 30 to 35% silver nanoparticles in tripropylene glycol monomethyl ether (Aldrich). Single core wires were connected to the ends of the printed contacts using a small amount of silver conductive paste (RS pro). A blank device was prepared by printing contacts directly onto unmodified glass. After the testing of the blank device a small amount of RS pro silver conductive paste was applied vertically through the interdigitated contacts. Lithographically-defined interdigitated gold contacts with a 15 µm electrode spacing were also coated with an SnS film prepared in the same way

Materials Characterisation

Transmission electron microscopy (TEM) was performed using a FEI Tecnai G2 20 with a LaB6 source operating at a 200 kV acceleration voltage. Selected area electron diffraction (SAED) in figure 1 (f) utilised a selected area aperture of 200 nm effective diameter. Crystal visualisation and SAED pattern indexing was performed using VESTA structural analysis software and SnS structure provided by the inorganic Crystal Structure Database entry no. 2437653. Scanning electron microscopy was performed using a Zeiss sigma scanning electron microscope. The relative surface coverage of the films from SEM and TEM was calculated using image thresholding on ImageJ, an area of film was chosen at the centre of the substrate for SEM. Raman was performed on a Renishaw 1000 Raman spectrometer with a 514 nm argon ion laser, powder X-ray diffraction (pXRD) was performed on a PANalytical X'pert modular powder diffractometer for bulk SnS with grazing incidence x-ray diffraction used for the SnS film. Atomic force microscopy (AFM) of the film was performed on a JPK nanowizard AFM using TESPA V2 tips in tapping mode, a line was scored through the film using a scalpel to obtain the film thickness with several height profiles being taken to obtain the average value. The individual sheet measurements were performed using a Bruker multimode with ScanAsyst HR tips. Ultra violet-visible (UV-Vis) spectroscopy was performed on a UV1800 shimadzu UV spectrophotometer using quartz cells with a 10 mm path length.

Characterisation of SnS Nanosheets From LPE by AFM and Electron Microscopy

Nanosheets were produced via the method of Brent et al.[1] Figure S1 (a) and (b) show a range of nanosheet thicknesses with an average thickness of 23.9 nm ± 15.6 nm (standard deviation). Figure S1 (c) and (d) shows an average sheet length of 224 nm ± 109.9 nm (s.d.), the high aspect ratio is present for exfoliated 2D materials due to the weak out of plane van der waals bonds[2]. This phenomenon has also been observed for liquid phase exfoliated SnS previously[1,3]. The sheets appear to have a high aspect ratio of lateral length to width due to anisotropy in the sheet plane[2] the average length measured was 2.03 times the width for the nanosheets measured via TEM which has been found previously by Brent and co-workers[1]. The average sheet thickness is slightly above the range expected from previous work on liquid phase exfoliated SnS. (7.8 nm +/- 2.5 nm for SnS exfoliated over 180 *g*). The average thickness is suspected to be skewed to a higher value due to the additional centrifugation step potentially removing thinner sheets[1]. Figure 1 (g) shows a plane spacing value of 2.78 Å which corresponds closely with the 2.92 Å spacing obtained for the (011) plane from the SAED. The plane spacing values obtained from SAED all match up well with those obtained from crystallographic data (supplementary table 1).[1] Slight differences in the HRTEM and SAED values obtained for may be due to slight calibration error or a small degree of sample tilt off axis.

Characterisation of blank and reference devices

The blank, bridged and photodetector devices were tested between -40 V and 40 V to produce the IV curves, this was performed under darkness and light for each. Each device was also placed under a + 40 V bias with intermittent 30s off 30s on illumination. The Langmuir- Blodgett device was also tested at a range of different voltages. The devices were tested using an oriel sol3A solar simulator at 1 sun illumination (1000 W m^{-2}), a keithley 2240 5 A source meter was used to supply the bias and measure the response. A uniblitz model VCM-DI shutter driver, es6b shutter and TTI TG315 function generator were set up to provide a fixed illumination and darkness time.

Noise measurements of the blank and Langmuir-Blodgett device were performed by monitoring the noise output of a SR865A lock-in amplifier. The reference frequency was swept using an Agilent 33220A arbitrary function generator. The measurement was performed inside a shielded box to minimise pick-up.

Monochromatic illumination tests were performed using a Bentham IL1 10 W halogen lamp, a TMc300 single monochromator and a Keithley 2636B SMU. A uniblitz model VCM-DI shutter driver, es6b shutter and TTI TG315 function generator were set up to provide the dark/ light cycle. A power detector with a 5 mm diameter opening was used to determine the power of each monochromatic wavelength.

The halogen lamp intensity spectra was obtained via a FDS1010 reference cell in order to obtain the monochromatic illumination intensity.

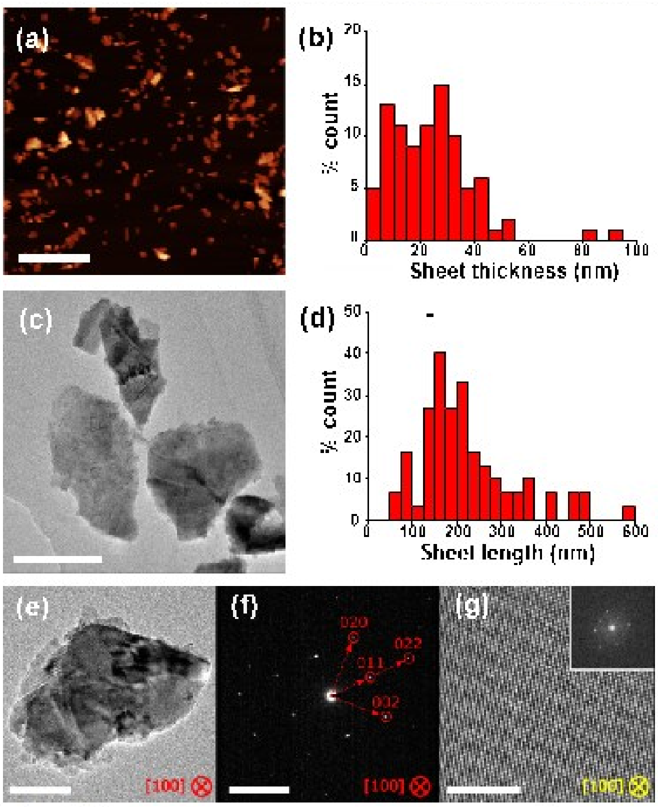

Figure S1: (a) AFM image of newly exfoliated, pre film deposited SnS nanosheets, scale bar: 1 µm, z scale 0 – 160nm (b) Histogram of nanosheet thicknesses centrifuged between 180 *g* and 3040 *g*, n= 100 (c) TEM image of nanosheets centrifuged between 180 and 3040 *g* scale bar: 250 nm (n=72) (d) Histogram of newly exfoliated SnS nanosheet lengths centrifuged between 180 *g* and 3040 *g*, n= 72, (e) TEM image of SnS nanosheet, scale bar: 50 nm (f) SAED of nanosheet shown in (e) with spot indices and viewing access indicated, scale bar: 5 nm^{-1}. (g) Background filtered atomic resolution HRTEM of SnS sheet in (e) with reduced Fast Fourier Transform (FFT) inset showing good correlation with SAED in (f), scale bar: 5 nm.

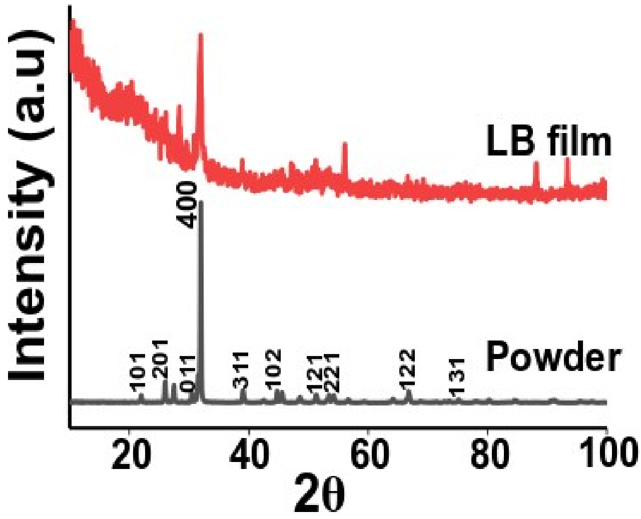

Figure S2: XRD with other assigned peaks

Table S2: SAED measured vs calculated plane distance values from crystallographic data

Plane	Measured value from SAED	Calculated from .CIF crystallographic data
(011)	2.92	2.93
(020)	1.99	1.99
(022)	1.45	1.47
(002)	2.14	2.16

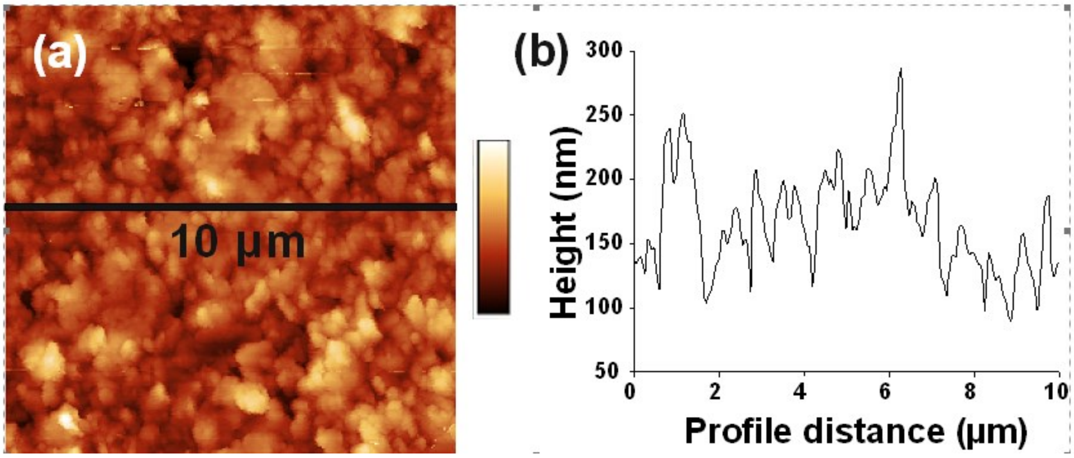

Figure S3: (a) AFM image taken from the centre of the film z scale 0 – 450 nm (b) roughness profile of SnS film

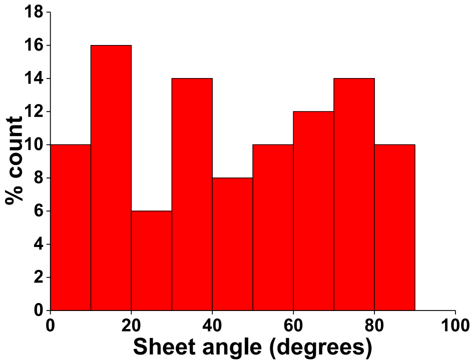

Figure S4: sheet angles taken from deposited film (n=50), horizontal direction taken as 0 degrees

Supplementary figure 4 shows a lack of preferred orientation of the SnS nanosheets upon deposition. The angles were determined from imagej software angle tool with the angle taken as the longest side of the nanosheet at an angle relative to the horizontal direction.

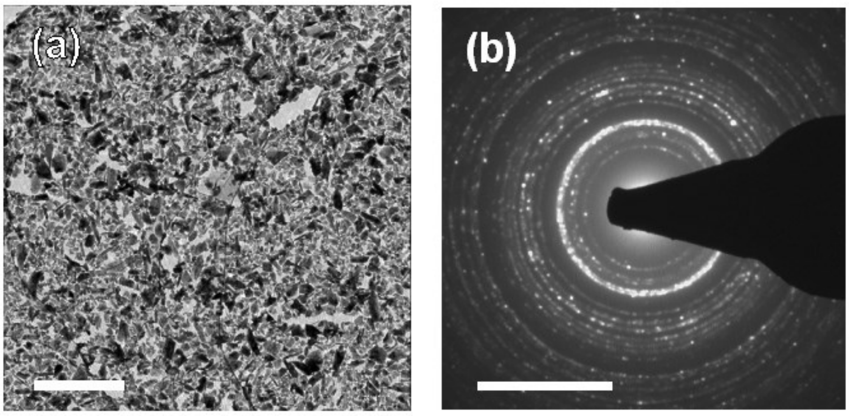

Figure S5: (a) TEM image of SnS film deposited onto a TEM grid, scale bar: 2 µm (b) TEM diffraction pattern of SnS film with indexed SAED pattern, scale bar: 5 nm^{-1}

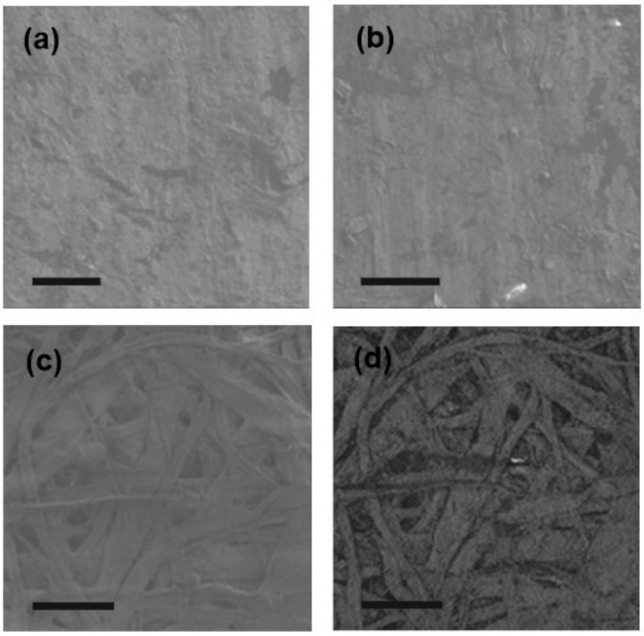

Figure S6: (a) SEM image of SnS coated onto parafilm 1.5 kV, SE, scale bar: 20 µm (b) SEM image of SnS coated onto aluminium foil, 1.5 kV, SE, scale bar 20: µm (c) Secondary electron SEM image of SnS coated onto filter paper, 1.5 kV, scale bar: 100 µm (d) backscatter imaging of SnS coated onto filter paper, 5kV, scale bar: 100 µm

Supplementary figure 6 shows SnS successfully coating onto a variety of substrates, The coating percentage varies compared to Si/SiO_2 substrates with values of (a) 75.7%, (b) 67% and (c),(d) 63.27%. Coating onto a variety of different materials along with the successful proof of concept device obtained in figure 5 indicates a potential viability for the creation of flexible devices.

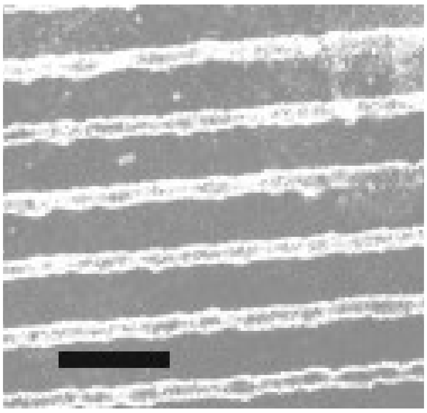

Figure S7: optical microscopy image of printed SnS contacts, scale bar 500 µm

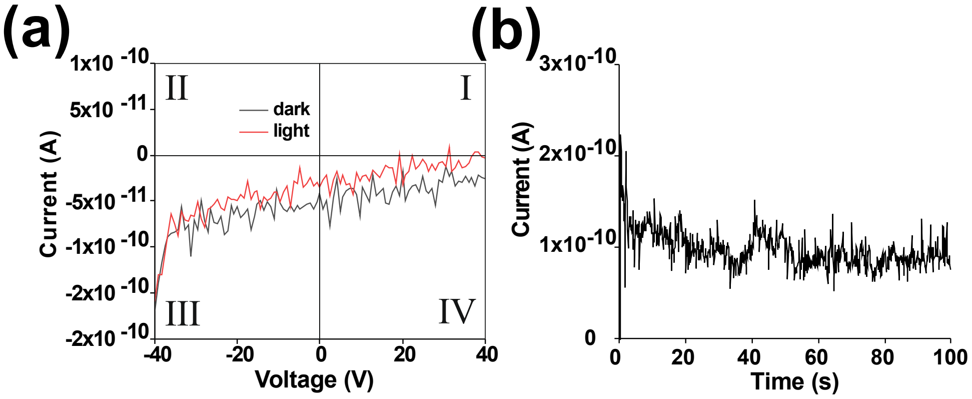

Figure S8: (a) IV curve of printed Ag interdigitated electrode on a cleaned substrate without a deposited SnS film under darkness and illumination (b) Same device under 40 V bias under fixed darkness/ illumination cycle.

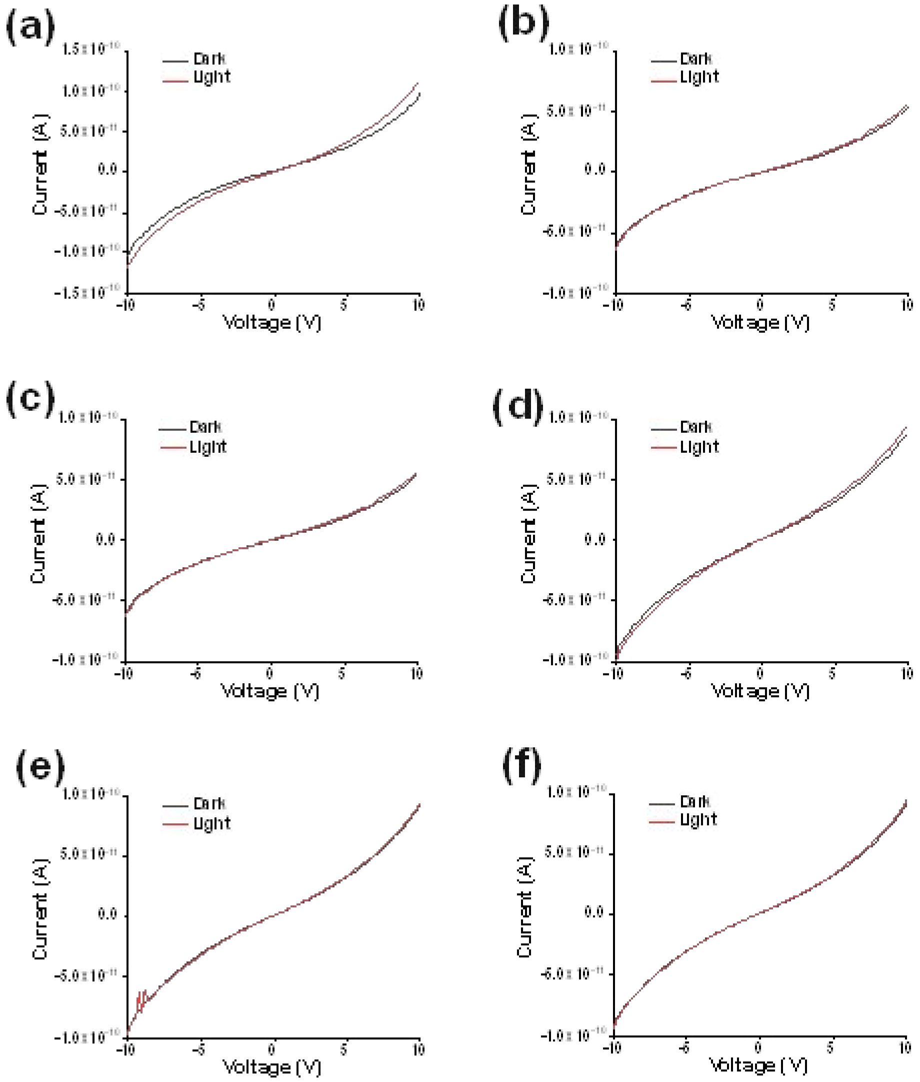

Figure S9 IV sweeps of interdigitated chip device under 350 nm, 405 nm, 500 nm, 600 nm and 800 nm (a), (b), (c), (d), (e) respectively, corrected for hysteresis

A lower response was found for longer wavelengths, as shown previously for IV curves obtained from SnS photoconductor type photodetectors. A higher response was previously observed for 380 nm at 31 mW cm^{-2} than for 850 nm at 55 mW cm^{-2} [4]. This may be due to SnS having a higher absorbance at shorter wavelengths.[1] There may also be an effect of higher frequency illumination generating a higher photoresponse.

The subsequent curves after 350 nm appear to exhibit only a small photoresponse, The currents generated at + 10 V are: 1.72×10^{-11} A, 2.6×10^{-12} A, 6.78×10^{-12} A, 3.3×10^{-13} A and 2.2×10^{-12} A for (a), (b), (c), (d), (e), (f) respectively. The intensities incident on the device were calculated with regards to a reference

The intensities obtained were: 0.255 µW cm^{-2}, 0.132 µW cm^{-2}, 0.307 µW cm^{-2}, 0.556 µW cm^{-2}, 0.984 µW cm^{-2} and 3.726 µW cm^{-2} for 350 nm, 405 nm, 450 nm, 500 nm, 600 nm and 800 nm respectively.

However, due to the small photoresponse the longer wavelength responses were tentatively assigned. The low monochromatic photocurrent response is expected due to the low responsivity of the device.

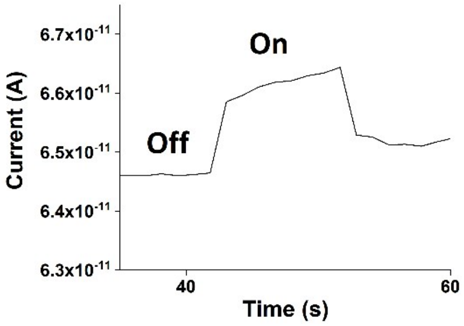

Figure S10 Single off/ on 405 nm monochromatic illumination cycle

…

References:

1. J. R. Brent, D. J. Lewis, T. Lorenz, E. A. Lewis, N. Savjani, S. J. Haigh, G. Seifert, B. Derby and P. O'Brien, *J. Am. Chem. Soc.*, 2015, **137**, 12689-12696.
2. J. Shen, Y. He, J. Wu, C. Gao, K. Keyshar, X. Zhang, Y. Yang, M. Ye, R. Vajtai, J. Lou and P. M. Ajayan, *Nano Lett.*, 2015, **18**, 5449-5454.
3. W. Huang, Z. Xie, T. Fan, J. Li, Y. Wang, L. Wu, D. Ma, Z. Li, Y. Ge, Z. N. Huang, X. Dai, Y. Xiang, J. Li, X. Zhu and H. Zhang, *J. Mater. Chem. C*, 2018, **10**, 9582-9593.
4. M. S. Mahdi, K. Ibrahim, A. Hmood, Naser M. Ahmed, S. A. Azzez and F. I. Mustafa, *RSC Adv.*, 2016, **6**, 114980-114988.
